# Management of patulous Eustachian tube dysfunction following bariatric surgery: a case report

**DOI:** 10.3389/fsurg.2025.1614291

**Published:** 2025-08-20

**Authors:** Amr Youssef Arkoubi

**Affiliations:** Department on Anesthesia and Surgery, Faculty of Medicine, Imam Mohammad Ibn Saud Islamic University (IMSIU), Riyadh, Saudi Arabia

**Keywords:** patulous Eustachian tube, bariatric surgery, autophony, balloon dilation, fat grafting

## Abstract

Patulous Eustachian Tube (PET) dysfunction is a rare condition characterized by an abnormally open Eustachian tube, leading to symptoms such as autophony, auditory fullness, and pulsatile tinnitus. This case report describes a 48-year-old female weighing 72.4 kilograms who developed persistent autophony and hearing her own breathing and heartbeat sounds following significant weight loss after sleeve gastrectomy. Initial treatments, including bilateral ventilation tubes, provided minimal relief. Diagnosis of PET was established through clinical and nasopharyngeal examination in the ear, nose and throat (ENT) department, revealing bilaterally open Eustachian tubes with a pronounced defect on the left ear. Surgical management included removal of the ventilation tubes, bilateral balloon dilation of the Eustachian tubes, and fat injection with plasma into the tubal opening (medial), utilizing fat harvested via liposuction. The patient was observed to have stable condition when her weight is above 80 kilograms, but the symptoms recurred while being under 80 kilograms, therefore her PET dysfunction was weight dependent. Postoperative outcomes showed temporary symptomatic relief lasting six months, with partial symptom recurrence. This case highlights the role of combined surgical interventions in managing PET and underscores the need for further research to develop durable treatment solutions for this challenging condition.

## Introduction

The Eustachian tube is a flexible, thin, epithelial-lined passage that links the middle ear to the lateral wall of the nasopharynx. The medial two-thirds of the tube consist of fibrocartilage, while the outer one-third is osseous (1). It remains closed at rest, offering the middle ear equilibrium, integrity, and safeguarding against nasopharyngeal secretions. It opens solely during mastication, yawning, and sneezing, facilitated by the salpingopharyngeus, tensor tympani, levator, and tensor veli palatini muscles. Conversely, Ostmann's fat pad, a conical mass of adipose tissue situated on the inferomedial portion of the cartilaginous section, facilitates tube closure and prevents the reflux of secretions (physiological valve) (2). The Eustachian tube has several functions, involving fluid elimination, defense of the middle ear from excessive noise or infections, ventilation, and adjustment of intratympanic pressure with the external auditory canal. The failure to perform the aforementioned steps is termed Eustachian tube dysfunction (3).

The patulous Eustachian tube (PET) was initially documented in 1864, when Schwartze noted the eardrum's synchronized movement with respiration (4, 5). The disorder is characterized by an abnormal openness of the Eustachian tube, leading to autophony, which is the perception of one's own voice and breathing sounds amplified in the ear. Other symptoms encompass auditory fullness and pulsatile tinnitus, albeit their presence is not stable. PET is believed to arise from the inefficiency of the tubal physiological valve resulting from an error in the lumen of the cartilaginous tube, which eventually hinders full closure (5). The development of PET may be attributed to the reduced size of a fat pad known as Ostmann's fat, located between the tensor veli palatini and the Eustachian tube opening (6, 7). A reduction in the amount of tissues surrounding the Eustachian tube may lead to diminished elasticity and compliance, hindering its closure due to the pressure from adjacent tissues (8, 9).

These characteristics may be present in post-bariatric patients, and current research suggests that the prevalence of PET following bariatric surgery is greater than that in the general public. Substantial and fast weight reduction (such as post-bariatric surgery) may result in a reduction of the soft tissue encasing the auditory tube, potentially increasing the chance of developing PET (10). PET individuals may be asymptomatic or exhibit symptoms ranging from moderate to severe. The predominant complaints include autophony (the subjective perception of hearing one's own voice while speaking), auditory fullness, tinnitus, and the perception of one's own breathing. The supine position, with the head positioned between the knees, upper airway infections, or the execution of the reverse Valsalva maneuver serve as factors for healing. The exacerbating factors include the practice of workouts, prolonged vocal usage, and the consumption of nasal or oral decongestants (10). PET dysfunction is diagnosed through a combination of clinical history, physical examination, and objective testing. Patients typically report autophony, aural fullness, and symptoms that worsen in upright positions but improve when lying down. Key diagnostic methods include tympanometry with respiratory monitoring, which reveals tympanic membrane movement synchronized with breathing, and nasopharyngoscopy, which directly visualizes an abnormally open Eustachian tube. Additional bedside tests, such as the Toynbee test and Valsalva maneuver, help differentiate PET from other forms of Eustachian tube dysfunction. Positional changes and nasal saline instillation may provide temporary symptom relief, further supporting the diagnosis. Advanced imaging, such as magnetic resonance imaging or computed tomography, is reserved for cases where anatomical abnormalities are suspected. A comprehensive evaluation using these tools ensures an accurate diagnosis and helps guide appropriate treatment strategies (11).

## Case presentation

A 48-year-old female weighing 72.4 kilograms presented with persistent autophony characterized by hearing her own heartbeat, breathing, and chewing sounds, which began a few months after undergoing sleeve gastrectomy. The symptoms were not associated with vertigo, tinnitus, or significant hearing loss. Previous interventions, including multiple medical consultations and the placement of bilateral ventilation tubes (permanent in the right ear and temporary in the left), provided minimal relief. Examination in the ENT department revealed bilateral ventilation tubes, clear nasal cavity and sinuses, and widely open Eustachian tubes, more pronounced on the left side. A diagnosis of Eustachian Tube dysfunction with patulous tubes was established. Superior semicircular canal dehiscence (SSCD) and other mimicking conditions were ruled out through clinical reasoning or imaging when indicated. The patient was observed to have stable condition when her weight was above 80 kilograms, but the symptoms recurred while being under 80 kilograms, therefore her PET dysfunction was weight dependent. The proposed management included removal of the ventilation tubes, bilateral balloon dilation of the Eustachian tubes, and fat injection with plasma into the Eustachian tube region using fat harvested via thigh liposuction. Surgical management involved three key steps: (1) removal of the previously placed ventilation tubes, (2) bilateral balloon dilation of the Eustachian tubes to restore tubal compliance and assess patency under pressure, and (3) targeted fat injection augmented with autologous plasma. Fat was harvested from the patient's thigh using standard liposuction under sterile conditions. The lipoaspirate was processed via centrifugation at 3000 rpm for 3 min to separate viable adipose tissue from oil and blood residues. Approximately 1.5–2 ml of purified fat was mixed with autologous platelet-rich plasma (PRP) in a 4:1 ratio to enhance graft survival. Under endoscopic guidance, the fat-PRP mixture was injected submucosally into the inferomedial wall of the Eustachian tube opening via a transnasal route, with care taken to avoid overcorrection or intraluminal deposition. Bilateral injections were performed, with emphasis on the left side where the tubal defect was more pronounced. Preoperative investigations, including coagulation profile and complete blood count, were unremarkable. The surgical procedure was completed successfully without intraoperative complications. Postoperative instructions included a course of antibiotics (amoxicillin/clavulanic acid), analgesics, nasal decongestants, and return to a regular diet, with a follow-up scheduled after one month. At the two-month follow-up, the patient reported a notable reduction in autophony, though mild symptoms persisted in the left ear. Counseling regarding the potential for recurrence and re-injection was provided. Symptomatic improvement was maintained for approximately six months postoperatively, after which the patient reported gradual recurrence of symptoms. This recurrence, though less severe, suggests the resorption of injected fat and highlights the potential need for repeat interventions to maintain long-term symptom relief. At the one-year follow-up, the patient continued to experience intermittent mild autophony, but overall quality of life had improved compared to the preoperative baseline. Plans for future reassessment and consideration of a second injection were discussed based on symptom progression.

## Discussion

Eustachian tube dysfunction encompasses two primary types with distinct pathophysiologies and clinical presentations: obstructive and patulous. Obstructive Eustachian tube dysfunction, the more common form, results from failure of the Eustachian tube to open properly, leading to negative middle ear pressure, conductive hearing loss, and aural fullness. In contrast, PET syndrome is characterized by an abnormally open tube, either continuously or intermittently, allowing abnormal sound transmission of the patient's own voice, breathing, or heartbeat—referred to as autophony. This open state disrupts middle ear pressure regulation and impairs auditory perception. The present case exemplifies true PET, given the hallmark symptoms of autophony and respiratory-synchronous tympanic membrane motion confirmed via nasopharyngeal endoscopy. Differentiating between these forms of Eustachian tube dysfunction is crucial, as treatment strategies diverge significantly; surgical interventions such as balloon dilation and tissue augmentation are often reserved for patulous forms, whereas obstructive cases typically benefit from pressure equalization strategies or allergy control. Clinician awareness of these distinctions is essential to avoid misdiagnosis and to appropriately counsel patients regarding prognosis and treatment options (12).

In the literature, there are limited accounts of clinical cases associated with substantial weight loss accompanied with the emergence of PET. Patients with PET experience symptoms including auditory fullness and autophony of voice or respiratory sounds resulting from an excessively open Eustachian tube (13, 14). The primary cause of PET is weight reduction (1, 15). In 1964, Pulec et al. (16) determined that the likely etiological factor for defective Eustachian tube patency in their study was attributed to weight reduction in 15 instances.

Pascoto et al. (17) indicated that symptoms of tubal dysfunction following bariatric surgery, including autophony, did not exhibit alterations in objective measurements from audiometry and tympanometry. Regrettably, the objective identification of tubal dysfunction remains challenging, as symptoms may not consistently manifest throughout the examination (e.g., tympanometry and Eustachian tube function tests). The diagnosis is contingent upon the patient's history and clinical manifestations. Due to the lack of objective diagnostic techniques for Eustachian tube dysfunction, the diagnosis frequently relies on clinical history (18). The predominant complaint among patients with Eustachian tube dysfunction is autophony, which adversely impacts their quality of life (19, 20). Autophony is frequently considered indicative of Eustachian tube dysfunction. Certain authors determined that this symptom is generic and may be attributed to various different illnesses, including superior canal dehiscence. Foreign objects in the eardrum may simulate autophony, resulting in an amplified sense of one's voice (1).

Munoz et al. (20) demonstrated a significant prevalence (21.28%) of PET among their patients. Autophony was observed in 96.6% of patients diagnosed with PET. Patients without PET achieved an average weight loss of 39.54 kg over 16.59 months, whereas patients with PET saw an average weight loss of 48.63 kg over 12.11 months. They asserted that the incidence of PET correlated with the rate and extent of weight reduction (20).

In 2009, Alhammadi et al. (19) documented the inaugural instance in English literature of a postoperative pulmonary embolism following laparoscopic Roux-en-Y gastric bypass surgery for obesity. A 44-year-old woman was referred for evaluation due to complaints of autophonia and auditory fullness. She began to see signs of PET around three months post-operatively after losing about 20 kg.

Letti et al. (21) assessed a cohort of eight patients who followed a restrictive diet, resulting in an average weight loss of 15 kg over 45–65 days, and had tubal dysfunction. The reduction of subcutaneous adipose tissue increases the permeability of the auditory tubules, as shown by researchers in a study examining the impact of a restrictive diet on individuals experiencing substantial weight loss, inadequate nutrition, and declining overall health.

Yoshida et al. (6) discovered that their investigation revealed a considerably reduced size of the connective tissue, particularly Ostmann's fatty tissue, surrounding the Eustachian tube in patients with PET. Acute weight loss may be associated with the loss of Ostmann's fatty anatomical structure in relation to patulous Eustachian tube. This observation parallels that of Poe et al. (4, 22), who conducted nasopharyngeal endoscopy of the Eustachian tube lumen and hypothesized that a PET results from the depletion of adipose tissue in the cartilaginous segment of the Eustachian tube.

Mucous-thickening agents and topical irritants (e.g., Lugol-gel, Bezold powder, or sodium iodide) that provoke mucosal edema near the pharyngeal opening may provide temporary relief from aural symptoms. Furthermore, the application of a paper patch to the tympanic membrane has been suggested. In certain PET instances when auditory fullness is the predominant symptom, the use of a 5 mm circular steri strip on the posterior-superior quadrant of the tympanic membrane has demonstrated efficacy (23, 24). In this instance, fat injection from the thigh was employed, resulting in a degree of improvement despite the noted recurrence.

Injection materials comprise fat, cartilage, calcium hydroxylapatite, and polydimethylsiloxane. This treatment is beneficial due to its minimally invasive nature and relative ease of execution. Numerous procedures are frequently necessitated by the absorption of the injected materials (11). Sudhoff et al. (25) evaluated the efficacy of a soft-tissue bulking agent by contrasting several Eustachian tube augmentation techniques: transpalatinal Eustachian tube augmentation performed under local and general anesthesia against augmentation with velotraction conducted under general anesthesia. The study concluded that transpalatinal Eustachian tube augmentation performed under local anesthetic yielded statistically significant clinical improvement. This enhancement can be ascribed to the intraoperative “feedback” obtained from patients under local anesthetic in an upright position, hence diminishing the necessity for recurrent interventions (25). Eustachian tube dilatation is often performed before fat injection in the management of PET dysfunction to optimize the success of treatment through restoring normal Eustachian tube function before permanent augmentation, enhancing fat graft stability and integration, minimizing the risk of overcorrection, and assessing the severity and reversibility of PET dysfunction.

While the diagnosis of PET in this case was based on clinical symptoms and direct visualization via nasopharyngoscopy, additional objective tests such as long-time-based tympanometry, nasal audiometry, and sonotubometry could have provided further confirmation and functional characterization. Long-time tympanometry is particularly useful in PET, as it allows for detection of tympanic membrane compliance changes over time, often synchronized with respiration. Nasal audiometry can detect the presence and intensity of autophonic symptoms by measuring sound conduction through the nasal cavity into the ear. Sonotubometry, which involves transmitting a sound into the nose and recording its passage through the Eustachian tube to the ear canal, offers a non-invasive assessment of tubal patency. These diagnostic tools are not only valuable for confirming the diagnosis but also for monitoring the effects of surgical intervention and guiding follow-up decisions.

The inclusion of balloon dilation as part of the surgical intervention in this case warrants further discussion, as its role in PET is controversial. Balloon dilation has shown promising results in obstructive Eustachian tube dysfunction by mechanically widening a constricted cartilaginous segment and improving tubal ventilation. However, in PET, where the tube is already abnormally open, dilating the lumen may theoretically worsen the condition or offer little benefit. In the current case, the rationale for balloon dilation was to assess the functional mobility of the Eustachian tube walls and to condition the tissue prior to fat injection, with the intent to optimize the integration and effectiveness of the augmentation material. Despite this theoretical benefit, the recurrence of symptoms within six months suggests that the contribution of balloon dilation to long-term relief was limited or perhaps unnecessary. Further research is needed to clarify whether pre-injection ballooning has any synergistic effect in PET cases or if its use should be avoided altogether in this patient subset.

## Conclusion

This case emphasizes the challenges in diagnosing and managing Patulous Eustachian Tube dysfunction, particularly in patients experiencing rapid weight loss. While combined surgical techniques, including balloon dilation and fat injection, offer temporary symptomatic relief, recurrence remains a concern. The findings underscore the importance of individualized management plans, patient education, and close monitoring. Further studies are warranted to explore innovative materials and techniques for sustained outcomes and to refine the diagnostic criteria for PET in post-bariatric surgery patients.

## Data Availability

The original contributions presented in the study are included in the article/Supplementary Material, further inquiries can be directed to the corresponding author.
